# The impact of pericardial disruption on heart function and remodeling after myocardial infarction in mice

**DOI:** 10.1007/s10554-026-03688-8

**Published:** 2026-03-21

**Authors:** Sara Munk Laursen, Ditte Gry Ellman, Charlotte Harken Jensen, Ditte Caroline Andersen

**Affiliations:** 1 Andersen Group, Department of Clinical Biochemistry, Odense University Hospital, Odense, Denmark; 2 Clinical Institute, University of Southern Denmark, Odense, Denmark

**Keywords:** Myocardial infarction, Mice, Vevo F2, Intact pericardium, Ruptured pericardium

## Abstract

Myocardial infarction (MI) remains a leading cause of deaths globally. Thus, reliable and relevant animal MI models are crucial to understand underlying disease mechanisms and develop new therapies. Generally, MI in rodents is induced by ligation of the left anterior descending artery (LAD) leaving a ruptured pericardium. This contrasts MI in man, where the pericardium remains intact. Herein, we therefore determined in adult mice whether pericardial disruption along MI influences left ventricular (LV) heart function, remodeling, and scar formation. Baseline heart function was assessed in 30 C57BL/6J adult, female mice, using state-of-the-art ultra-high-resolution ultrasound imaging (Vevo F2). After one week, MI was randomly introduced by LAD in two groups of mice with or without rupturing the pericardium. Functional heart recovery was evaluated 1- and 10 weeks post-MI, whereas success of MI introduction, animal survival, and scarring were quantified at week 10 by Masson’s Trichrome histology and compared between groups. The success of MI introduction as well as mouse survival was equal between groups (*p* > 0.05). Cohort baseline data revealed normal heart function (55.9 ± 4.5% ejection fraction (EF), 33.3 ± 3.2% fractional shortening (FS), 23.3 ± 4.2 µL end-systolic volume (ESV), 52.5 ± 5.1 µL end-diastolic volume (EDV) and 29.2 ± 2.1 µL stroke volume (SV) (mean, SD, *n* = 21)). At 1-week post-MI, all mice exhibited significant (all *p* < 0.05) systolic dysfunction, which persisted at 10-weeks post-MI (46.8 ± 7.4%- vs. 49.3 ± 10.0% EF, 24.7 ± 6.3%- vs. 24.7 ± 5.6% FS, and 35.7 ± 12.8 µL- vs. 37.0 ± 14.9 µL ESV for intact and ruptured pericardium, respectively (mean, SD, *n* = 9 and 12)) with no major differences between groups (all *p* > 0.05). For ruptured pericardium, we did see a slight increase in EF and SV from week 1 to 10. Ventricular remodeling was equally evident in both groups, with increased (all *p* > 0.05) LV mass and end-systolic- and end-diastolic area, but no difference (all *p* > 0.05) between groups. A mild increase in LV posterior wall thickness was exclusively apparent for ruptured pericardium, whereas anterior wall thickness was similar between groups. In line, infarct size was similar between groups (*p* > 0.05). Pericardial integrity does not universally alter heart function and scar size following LAD ligation in adult female mice, but may have some previously unnoticed compensatory effects not present after MI in man and that should be considered when modeling MI in the mouse.

**Supplementary Information:**

The online version contains supplementary material available at 10.1007/s10554-026-03688-8.

## Introduction

Cardiovascular disease contributes to the largest proportion of mortality worldwide [1], with ischemic heart disease representing the leading cause of deaths globally [2]. Among ischemic heart disease, myocardial infarction (MI) is responsible for a large proportion of the cardiovascular-related morbidities and deaths [3–5]. Cardiac remodelling after MI includes a first phase of apoptosis and necrosis, followed by a phase of inflammation, matrix degradation, and fibroblast expansion, whereafter a final phase of collagen accumulation and scar maturation occurs [6–8]. Although the formation of a scar is an intrinsic mechanism necessary to prevent infarct expansion, excessive or prolonged fibrotic remodeling contributes to ventricular stiffening and dysfunction [9]. Modulating the process of fibrosis after MI is thus of particular interest to develop MI therapies. The pericardium, a supportive structure that surrounds and protects the heart while providing lubrication and less friction [10] has recently gained additional interest in relation to MI. Indeed, fate mapping studies have shown that part of the MI-remodelling cardiac fibroblasts are derived from the inner layer of the pericardium; the epicardium [11] and upon MI, will form collagen I-secreting myofibroblasts that aggravate cardiac fibrosis. Moreover, the pericardium itself appears to function as a paracrine organ involved in the fibrotic response after MI [6, 12].

To better understand MI and develop therapies, animal MI disease models have been developed [13–16]. In particular, surgical permanent ligation of the left anterior descending coronary artery (LAD) [13, 14, 17–20] is a widely accepted MI mouse-model, where irreversible cardiomyocyte necrosis occurs followed by remodeling with persistent scarring. This leads to contractile dysfunction, progressive left ventricular (LV) dilation, and thinning of the posterior ventricular wall [16]. However, despite similarities are high between MI in humans and the mouse LAD model, surgical techniques vary considerably and may affect heart function [13, 16, 18–24] and especially the pericardial rupture in the mouse LAD model contrasts the human MI setting, where the pericardium remains intact after MI. Since the pericardium not only serves as a mechanical support, but also provides a paracrine- and cellular reservoir affecting the MI process [25–28], this difference despite being controversial [29–31] may have a large impact on the remodeling process in the mouse MI model. To overcome this issue, Hassanabad and co-workers [28] modified the LAD procedure avoiding disruption of the pericardium, and observed improved animal survival and MI recovery, while echocardiography was used to assess ejection fraction (EF) and the success of MI [28]. Indeed echocardiography is the gold standard for heart function assessment in clinical practice [32], but it is also a vital non-invasive tool for visualizing cardiovascular structures and serial assessment of heart function in mice [33]. When using echocardiography, functional parameters such as EF, fractional shortening (FS), and stroke volume (SV) offer direct insight into the contractile performance of the LV, making them essential for evaluating systolic function post-MI. Among these, EF is particularly sensitive to changes in LV performance and is especially valuable to track recovery or progression of dysfunction [32]. On the other hand, FS complements EF by reflecting localized wall motion, whereas SV helps quantify the heart’s ability to maintain output under stress or remodeling. Finally, dimensional parameters provide knowledge on the heart’s structural context [32]. When interpreted together, these parameters allow for a more nuanced assessment of functional impairment post-MI [34]. Hassanabad et al., used the Vevo 2100 Echo-platform, but they did not perform any detailed evaluation whether pericardial integrity impacted neither cardiac function nor scar size [28]. Moreover, recent advances have led to the introduction of new high-resolution echocardiographic systems more suitable for small animal models [32, 33], where the Vevo F2 Echo-system has replaced the Vevo 2100 platform offering a broader range of ultrasound frequencies (71–1 MHz). This allows ultra-high-resolution imaging with sufficient depth for functional heart studies specifically in small sized animals like the mouse and even in zebrafish [35]. With these new imaging modalities, a better sensitivity and visualization of vasculature is gained [36], and ultrasound guided cardiac injections [37] and complex longitudinal studies may be accomplished [38]. Echocardiography may however still be limited by subjectivity of the operator, and standardized imaging protocols and guidelines are thus required [32, 33, 39–43].

Herein, we thus set out to longitudinally investigate the impact of pericardial disruption in the mouse LAD model on LV heart function utilizing the advanced Vevo F2 Echo-system and also to evaluate the effect on scar remodeling with an endpoint at 10 weeks, a timepoint where MI formation is well established [44].

## Materials and methods

### Mice

Female C57BL/6J mice (*n* = 30) were purchased from Janvier (Le Genest-Saint-Isle, France) and housed at the Biomedical Laboratory, University of Southern Denmark, in individually ventilated cages in a 12/12 light/dark cycle with free access to food and water. The experiments started with a baseline scanning at nine weeks of age. All animal experiments were approved by the Danish Council for Supervision with Experimental Animals (# 2022-15-0201-01119).

### Echocardiography

**Fig. 1:**
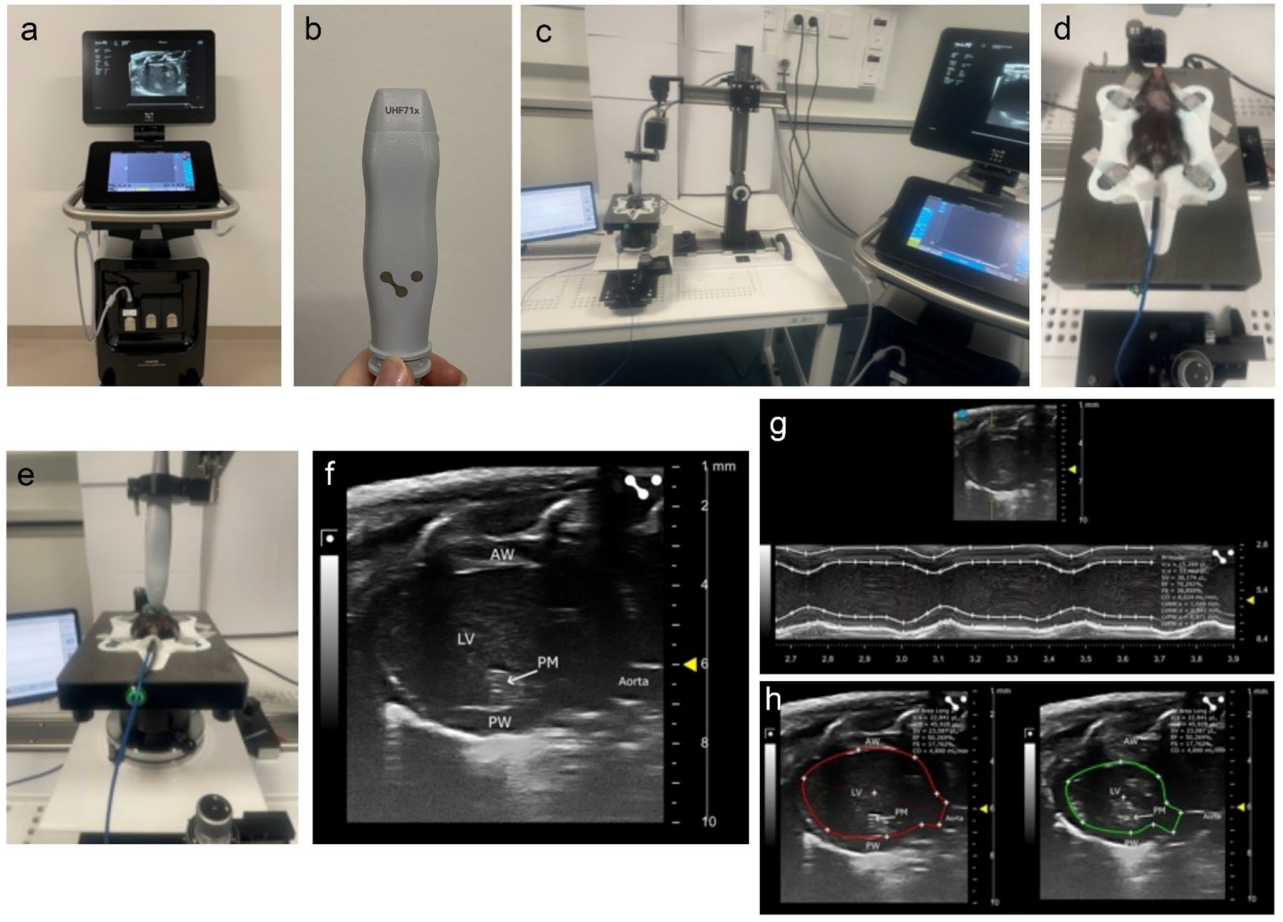
Experimental setup for murine echocardiography and representative images and measurements in PSLAX view, B- and M-mode. (**a**) Vevo F2 ultrasound imaging system. (**b**) UHF71x transducer. (**c**) Complete setup showing the Animal Monitoring System, prepared mouse, Integrated Rail System with transducer, and Vevo F2 ultrasound system. (**d**) Mouse in supine position on a pre-warmed platform with thorax shaved for scanning and paws connected to monitoring electrodes. (**e**) Transducer positioning for PSLAX view. Representative images of (**f**) B-mode image of the left ventricle in PSLAX view, and (**g**) M-mode image with left ventricular measurements in PSLAX view at mid-papillary level. (**h**) Endocardial tracings on B-mode images in PSLAX view, left: LV in end-diastole with tracing (red); right: LV in end-systole with tracing (green). LV: left ventricle, RV: right ventricle, AW: anterior wall, PW: posterior wall, PM: papillary muscles, PSLAX: parasternal long axis, PSSAX: parasternal short axis

High-resolution echocardiographic images were acquired using the Vevo F2 ultra-high-frequency, real-time ultrasound system (FUJIFILM VisualSonics Inc., Toronto, ON, Canada). The system was equipped with a 71 − 30 MHz center frequency linear array transducer (UHF71x, FUJIFILM VisualSonics Inc., Toronto, ON, Canada). To ensure consistent transducer positioning and reproducibility of imaging angles, the Vevo Integrated Rail System with Vevo Animal Monitoring System (FUJIFILM VisualSonics Inc., Toronto, ON, Canada) was utilized, providing reliable support for image acquisition (Fig. 1a-c).

Echocardiographic parameters were obtained using two imaging modalities: long-axis M-mode at the mid-papillary muscle level (FS, LV mass, and left ventricular anterior wall (LVAW) thickness, and left ventricular posterior wall (LVPW) thickness in end-diastolic, and end-systolic phases) and Modified biplane Simpson B-mode (EF, SV, and LV volume and area at average in end-diastolic, and end-systolic phases), in accordance with established small animal imaging guidelines [35, 45].

Prior to imaging, mice were anesthetized as described below. To facilitate optimal imaging conditions, all fur from the ventral thoracic area, extending from the lower ribcage to most of the upper ribcage, was carefully removed by shaving, ensuring clear imaging of the middle and left side of the chest.

Following preparation, mice were positioned supine on a pre-warmed imaging platform integrated within the Vevo Animal Monitoring System (Fig. 1d), and limbs were fixed with adhesive tape to ECG electrodes integrated into the animal platform with acoustic gel in-between. The latter enables monitoring of heart rate (HR) and respiration rate while core body temperature was measured via a rectal probe connected to the monitoring system. Acoustic gel was applied to the shaved thoracic area to enhance ultrasound transmission, and the transducer was carefully lowered into place using the Integrated Rail System.

Series of real-time echocardiographic images were acquired (Fig. 1e-g and Online Resource 1) in the parasternal long-axis (PSLAX) view (Fig. 1e-f). This method was selected based on established protocols for assessing LV heart function in adult mice [45]. Following these guidelines, we performed echocardiographic imaging of mice in PSLAX view, using B-mode and M-mode imaging to thoroughly obtain relevant measurements for evaluation of LV wall integrity and systolic function. The PSLAX view of the LV was obtained by positioning the transducer at the lower one-third of the chest, parallel to the central axis of the mouse body, and rotated counterclockwise by 15°–30°, keeping it close to the surface (Fig. 1e). Imaging in M-mode (Fig. 1g) and B-mode (Fig. 1h) were performed at: baseline (week − 1), and 1 week, and 10 weeks post-MI, to evaluate heart function post MI.

Similar echocardiographic images were obtained in parasternal short-axis (PSSAX) view (Online Resource 1), by rotating the transducer 90° clockwise from the PSLAX view, slightly tilted toward the apex to visualize the papillary muscles (PMs), ensuring an anatomically accurate short-axis view. M-mode measurements were likewise obtained in PSSAX view and complied with measurements in the PSLAX view (data not shown).

### Myocardial infarction

Mice were anesthetized with an intraperitoneal delivered mixture of xylazine (10 mg/kg) and ketamine (100 mg/kg), intubated, and ventilated. The mice were shaved on the thoracic area and fixed with adhesive tape exposing the thoracic area with the nose pointing away from the person performing the surgery. Following a left-sided thoracotomy the MI was performed as previously described [12]. Briefly, the LAD was ligated using an 8.0 Prolene suture either with intact or ruptured pericardium. For the last group, the pericardium was ruptured at the beginning of the surgery. Finally, the muscle layers were sutured with a 6.0 Prolene suture whereas the skin was closed with 5.0 Ethicon Vicryl. Post-surgery mice received subcutaneous injections of saline to prevent dehydration and analgetic treatment with the extended-release Buprenorphine Ethiqa XR (3.25 mg/kg) as recommended.

### Measurements, image analysis, and calculations

All echocardiographic examinations were conducted and analyzed by a single trained individual using Vevo LAB software version 5.8.2 (FUJIFILM VisualSonics Inc., Toronto, ON, Canada), blinded for the experimental groups. The Modified biplane Simpson’s method with 2D B-mode imaging was selected as the primary approach for assessing LV function and dimensions, due to its known accuracy in quantifying EF, end diastolic volume (EDV), and end systolic volume (ESV), especially in cases of regional wall motion abnormalities and altered LV geometry [33]. SV was calculated from volumetric measurements (EDV – ESV), providing a reliable alternative when Doppler data are unavailable [46]. Although M-mode imaging offers high temporal resolution for measuring FS and LVAW- and LVPW thickness, geometric assumptions of the LV limit its utility in post-MI remodeling. We therefore also exploited the Modified biplane Simpson’s method for consistent and comprehensive evaluation of the heart function.

### Histological staining and infarct size

At week 10 post-MI, mice were sacrificed by cervical dislocation, and the entire thorax encompassing the heart was quickly transferred to 10% neutral buffered formalin (NBF) for overnight fixation, to preserve the pericardium. The fixed heart was carefully dissected, stored in phosphate buffered saline (PBS) before dehydration and paraffin embedding. Paraffin embedded hearts were sectioned (10 μm) from the apex to the base in an accurate stepwise fashion, and sections were placed on KNITTEL StarFrost^®^ adhesive slides.

For confirmation of the presence of MI in the two groups of mice and to quantify MI remodeling as evaluated by infarct size, representative sections were stained with Masson’s trichrome as previously described [12]. In brief, sections were deparaffinized and rehydrated using Histoclear (x2) and decreasing concentrations of ethanol (3 × 99%, 2 × 96%, and 1 × 70%) before fixing in pre-heated Bouin’s solution. Sections were rinsed in running tap water, stained in Weigert’s iron hematoxylin, re-rinsed in running tap water, and then stained in Biebrich scarlet-acid fuchsin. Finally, sections were differentiated with phosphotungstic/phosphomolybdic acid before counter staining with aniline blue, followed by dehydration in increasing concentrations of ethanol (2 × 96%, and 2 × 99%), and mounting with Pertex^®^. The stained sections were imaged using a Leica MDG41 stereo microscope equipped with a Leica FLEXACAM C1 digital microscope camera. Imaging the entire specimen area was performed using the same settings including exposure and magnification. Images were analyzed in Qupath v0.6.0, where scar size was quantified as the collagen-rich (non-viable) myocardial area relative to the entire myocardial area for each individual section. This scar size was summarized across all transverse sections from each heart across groups [47]. Finally, the overall scar size for each group was calculated as area under the curve (AUC) and expressed as a percentage of collagen positive myocardium of the total myocardial area for all hearts within groups.

### Statistics

All statistical analyses were conducted using GraphPad Prism version 10.4.1 (GraphPad Software, La Jolla, CA). Group differences were assessed using two-way ANOVA with repeated measures for echocardiographic measurements and stepwise scar size, (percentage of fibrotic tissue relative to total myocardium). Post-hoc comparisons were conducted using Tukey’s multiple comparisons test. Scar size across groups was additionally quantified as the area under the curve (AUC), and group differences in AUC were assessed using a two-tailed unpaired t-test. The success of MI induction was evaluated using a chi-square test with Yates’ correction. Survival over the study period was compared between groups using the log-rank (Mantel–Cox) test. All quantitative data are presented as mean ± SD, and p-values ≤ 0.05 were considered statistically significant.

## Results

### Mouse survival after MI and the success of MI introduction were equal between mice with intact and ruptured pericardium

**Fig. 2:**
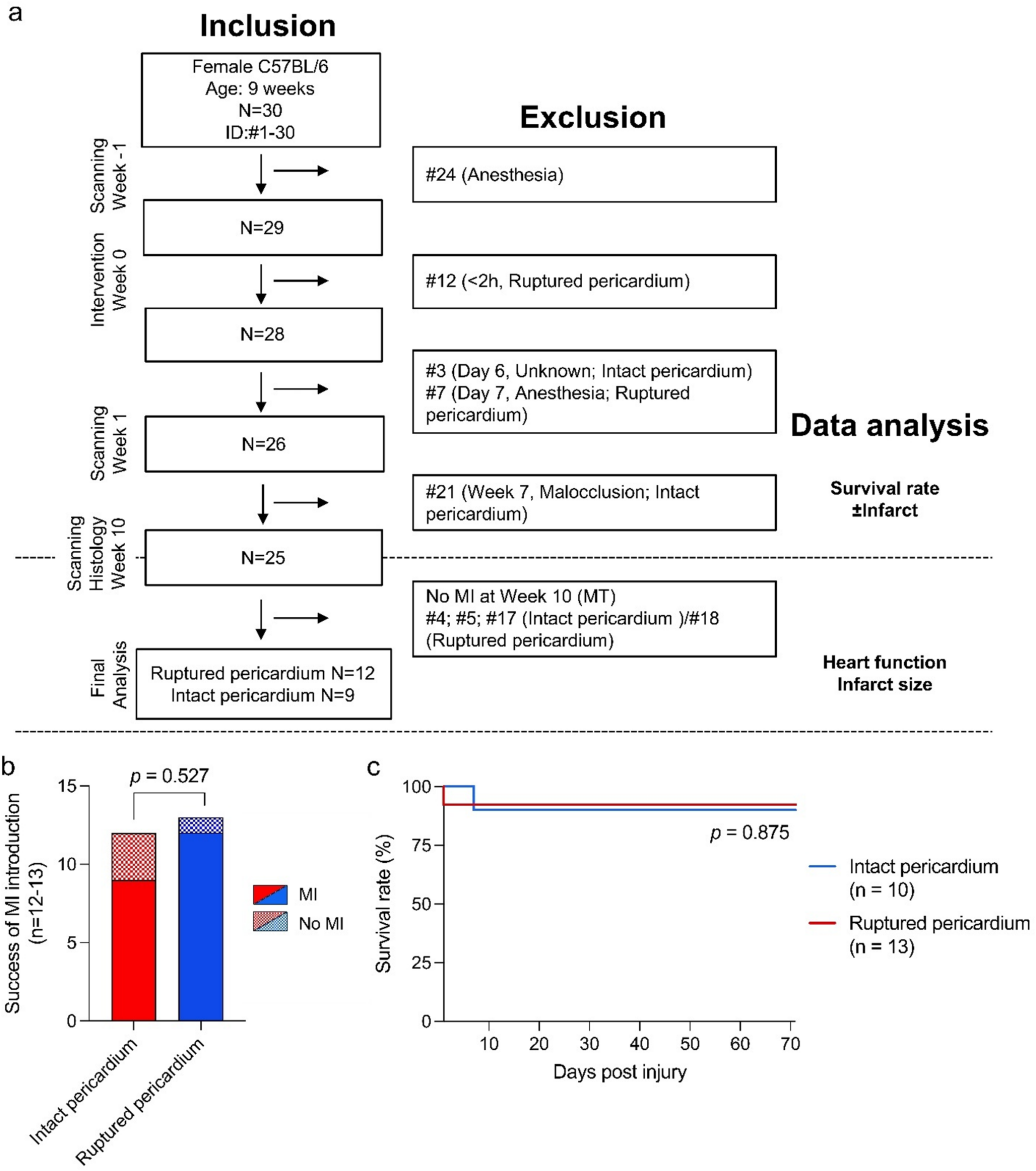
Study design and survival. (**a**) Overview of study timeline, inclusion and exclusion of mice and resulting analyses. (**b**) Success rate of MI induction in the groups with intact and ruptured pericardium (**c**) Survival of the mice during the study in the two groups with either intact or ruptured pericardium. MI: Myocardial infarction

A total of 30 female mice were included in the study at baseline (week − 1). One mouse (#24) died of anesthesia at baseline before echocardiography and another mouse (#12) died at intervention (week 0) within 2 h possibly due to a large MI (Fig. 2a). One mouse (#3) with intact pericardium died for unknown reason at day 6, whereas anesthetic complications during week 1 scanning for one mouse (#7) with ruptured pericardium resulted in death. Twenty-six mice were scanned at week 1, but one mouse (#21) was found dead at week 7 due to malocclusion (Fig. 2a), a known occasional phenotype seen in research mice [48]. Whereas mouse #3 and #12 were included in the survival assessment, five mice (#3, #7, #12, #21, #24) were excluded from the final study.

Thus, twenty-five mice underwent echocardiography at the 10-weeks endpoint and mice were hereafter sacrificed and dissected hearts were sectioned (Fig. 2a). Based on the absence of a visual MI using histology throughout the heart, three mice with intact- (#4, #5, #17) and one with ruptured (#18) pericardium were excluded from the final analysis as well (Fig. 2a). The success of MI introduction was equal between groups with intact- (9/12) and ruptured (12/13) pericardium (*p* = 0.527; Fig. 2b). Likewise, we did not observe a significant difference (*p* = 0.875) in mouse survival during the 10 weeks follow up (Fig. 2c) and at 10 weeks post-MI, survival was 90.0% and 92.3% for mice with intact- or ruptured pericardium, respectively. These data suggest that MI may be introduced with similar success using LAD with either intact- or ruptured pericardium.

### Heart function declined after MI independent of pericardial integrity, but compensatory mechanisms slightly occurred for mice with pericardial rupture

**Fig. 3:**
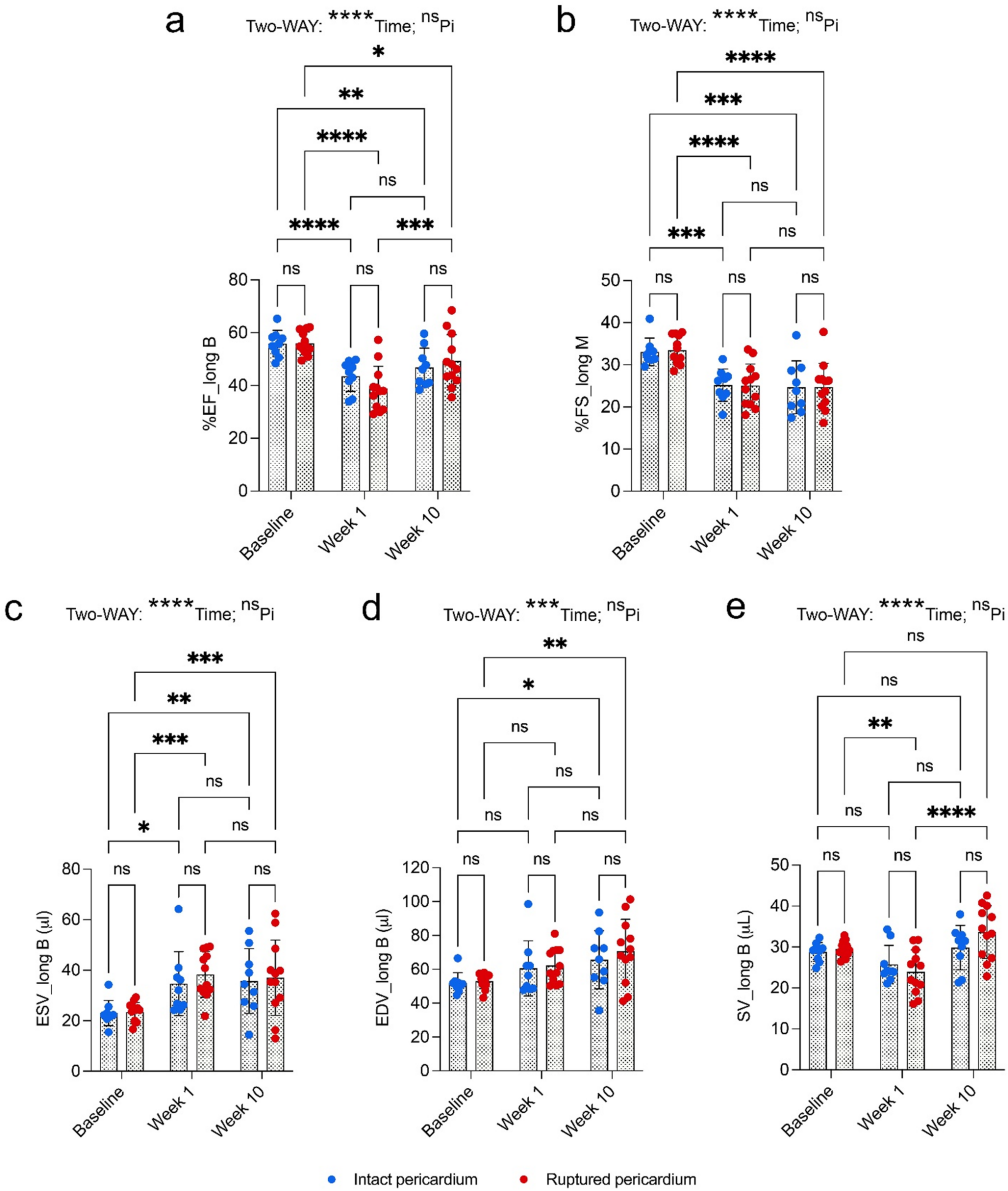
Left ventricular heart function analysis. Functional parameters (**a**) EF, (**b**) FS, and LV volumes (**c**) ESV, (**d**) EDV as well as hemodynamic parameter (**e**) SV, in mice with intact or ruptured pericardium at baseline, 1, and 10 weeks post-MI. EF: ejection fraction, FS: fractional shortening, LV: left ventricle, ESV: end-systolic volume, EDV: end-diastolic volume, SV: stroke volume. Pi: pericardial integrity, ns: non-significant. Statistics include two-way ANOVA with post hoc testing, *: *p* ≤ 0.05, **: *p* ≤ 0.01, ***: *p* ≤ 0.001, ****: *p* ≤ 0.0001

At baseline (week − 1), no difference (*p* > 0.05) was observed in EF, FS, ESV, EDV, and SV between mouse groups (Fig. 3a-e). As such, data showed that the healthy cohort of adult female mice anesthetized with Ketamine/Xylazine on average exhibited 55.9 ± 4.5% EF, 33.3 ± 3.2% FS, 23.3 ± 4.2µL ESV, 52.5 ± 5.1µL EDV and 29.2 ± 2.1µL SV (mean, SD, *n* = 21; Fig. 3a-e and data not shown). These data agree with previous results [33, 49–51] and validated the overall echocardiographic setup using the Vevo F2 system. After MI at week 0, hearts were longitudinally re-evaluated by echocardiography at 1- and 10 weeks post MI, showing an overall and expected reduction in heart function (Two-way ANOVA, *p* < 0.001 to *p* < 0.0001) (Fig. 3a-e and Online Resource 2). In specific at week 1, we found a significant decline from baseline in EF to 43.6 ± 5.9% (*p* < 0.0001) and 38.8 ± 8.6% (*p* < 0.0001), and in FS to 25.2 ± 3.8% (*p* = 0.0005) and 25.1 ± 5.1% (*p* < 0.0001), as well as an increase in ESV to 34.7 ± 12.6µL (*p* = 0.0153) and 38.4 ± 9.1µL (*p* = 0.0003) for intact- and ruptured pericardium, respectively (mean, SD, *n* = 9 and 12; Fig. 3a-c and data not shown). Notably, these three parameters are considered the best echocardiographic indicators for heart- dysfunction and remodeling post-MI [46] and the data thus emphasize that MI compromises heart function and -remodeling in both surgery schedules. However, there was no difference (*p* > 0.05) in EF, FS and ESV between MI hearts with intact- or ruptured pericardium (Fig. 3a-c). Pooling the data across groups revealed that the entire cohort of female mice one week post-MI exhibited an EF of 40.8 ± 7.7%, a FS of 25.1 ± 4.5%, an ESV of 36.8 ± 10.6µL, an EDV of 61.5 ± 12.8µL, and a SV of 24.7 ± 5.1µL (mean, SD, *n* = 21; Fig. 3a-e and data not shown). The heart dysfunction persisted for both groups at week 10 post-MI where the study was terminated (Fig. 3a-c). As such, we found an EF of 46.8 ± 7.4% and 49.3 ± 10.0%, a FS of 24.7 ± 6.3% and 24.7 ± 5.6%, and an ESV of 35.7 ± 12.8µL and 37.0 ± 14.9µL for intact- and ruptured pericardium, respectively (mean, SD, *n* = 9 and 12; Fig. 3a-c and data not shown).

Despite that there was no significant difference between MI hearts for intact- and ruptured pericardium (*p* > 0.05) in EF, FS and ESV, we did observe a slight 10.6 ± 8.0%, but significant (*p* = 0.0001) increase in EF from week 1 to week 10 for ruptured pericardium, whereas EF remained constant (3.2 ± 8.1% (*p* = 0.4379)) for intact pericardium (Fig. 3a and data not shown). Likewise, SV was significantly increased from week 1 to 10 for ruptured pericardium, exclusively (Fig. 3e). Thus, these data suggest comparable functional impairment of the hearts post-MI regardless of pericardial integrity, but minor compensatory mechanisms may exist for MI hearts with ruptured pericardium only.

### Except for posterior wall thickening in MI hearts with ruptured pericardium, overall ventricular remodeling was independent of pericardial integrity

**Fig. 4:**
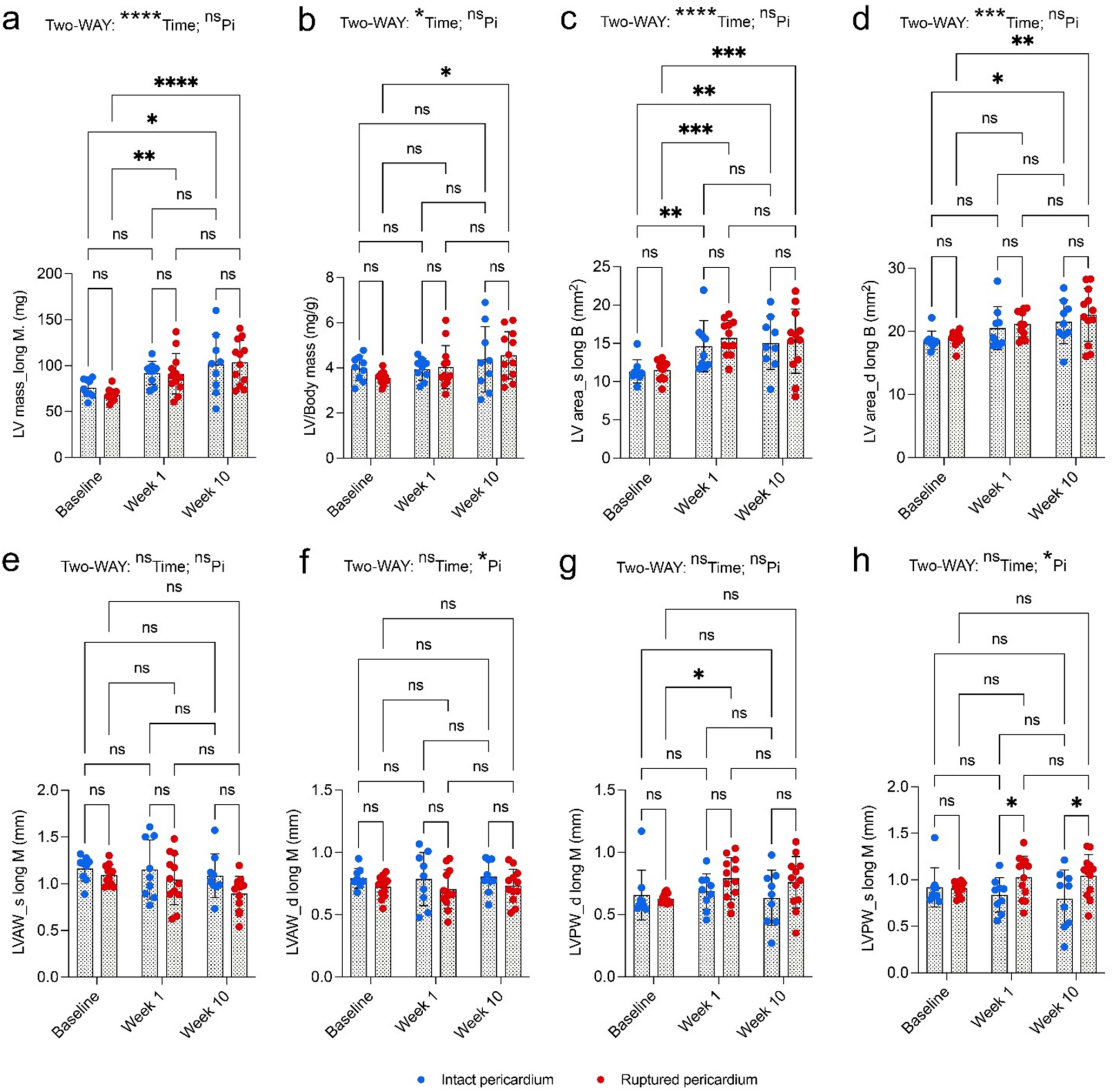
Left ventricular structural parameters over time. (**a**) LV mass, (**b**) LV/body mass ratio, (**c**) End-systolic- and (**d**) End-diastolic LV areas. End-systolic and end-diastolic LV wall thicknesses (**e-f**) LVAW and (**g-h**) LVPW respectively, in mice with intact or ruptured pericardium at baseline, 1, and 10 weeks post-MI. LV: left ventricle, AW: anterior wall, PW: posterior wall, Pi: pericardial integrity, ns: non-significant. Statistics include two-way ANOVA with post hoc testing, *: *p* ≤ 0.05, **: *p* ≤ 0.01, ***: *p* ≤ 0.001, ****: *p* ≤ 0.0001

LV hypertrophy as reflected by an increase in LV mass is a known compensatory mechanism post-MI [9, 52, 53]. Overall, LV mass was increased after MI (Two-way ANOVA, *p* < 0.0001), and already one week post-MI, we observed a significant (*p* = 0.0086) increase in LV mass from baseline in hearts with ruptured pericardium, and at 10 weeks post-MI, both intact- (101.2 ± 32.7 mg versus 75.7 ± 9.8 mg; *p* = 0.0137) and ruptured (103.8 ± 23.9 mg versus 67.9 ± 6.6 mg; *p* < 0.0001) pericardial MI hearts revealed higher LV masses as compared to baseline (Fig. 4a). Similar data was obtained when accounting for body size (Two-way ANOVA, *p* < 0.05), yet to a lesser extent (Fig. 4b).

We observed significant increases (Two-way ANOVA, *p* < 0.0001 and *p* < 0.001) in both end-systolic- ((intact: 15.1 ± 3.5 mm^2^ versus 11.3 ± 1.5 mm^2^; *p* = 0.003) and ruptured: (15.3 ± 4.2 mm^2^ versus 11.5 ± 1.2 mm^2^; *p* = 0.0005)) and end-diastolic- ((intact: 21.5 ± 3.5 mm^2^ versus 18.6 ± 1.5 mm^2^; *p* = 0.0344) and ruptured: (22.6 ± 4.2 mm^2^ versus 19.0 ± 1.1 mm^2^; *p* = 0.0015)) LV area post-MI for both groups, especially at week 10 (Fig. 4c-d) emphasizing LV remodeling. However, no differences (all *p* > 0.05) were observed for neither LV mass, LV mass/body weight, nor systolic- and end-diastolic area between the two groups at 10 weeks (Fig. 4a-d). The thickness of the LV anterior wall (LVAW) during end-systole and end-diastole was relative consistent between groups and throughout time (*p* > 0.05) (Fig. 4e-f). Whereas End-diastolic LVPW thickness remained constant (Fig. 4g), we did observe a significant difference in systolic LV posterior wall (LVPW) thickness at both 1- (*p* = 0.0488) and 10- (*p* = 0.0108) weeks post-MI between intact- and ruptured pericardium (Fig. 4h). This increased end-systolic LVPW thickness for mice with ruptured pericardium could represent a compensatory mechanism explaining the increased EF and SV observed at week 10 exclusively for ruptured pericardium (Fig. 3a + e).

### Scar size after MI was similar between intact- and ruptured pericardium

**Fig. 5:**
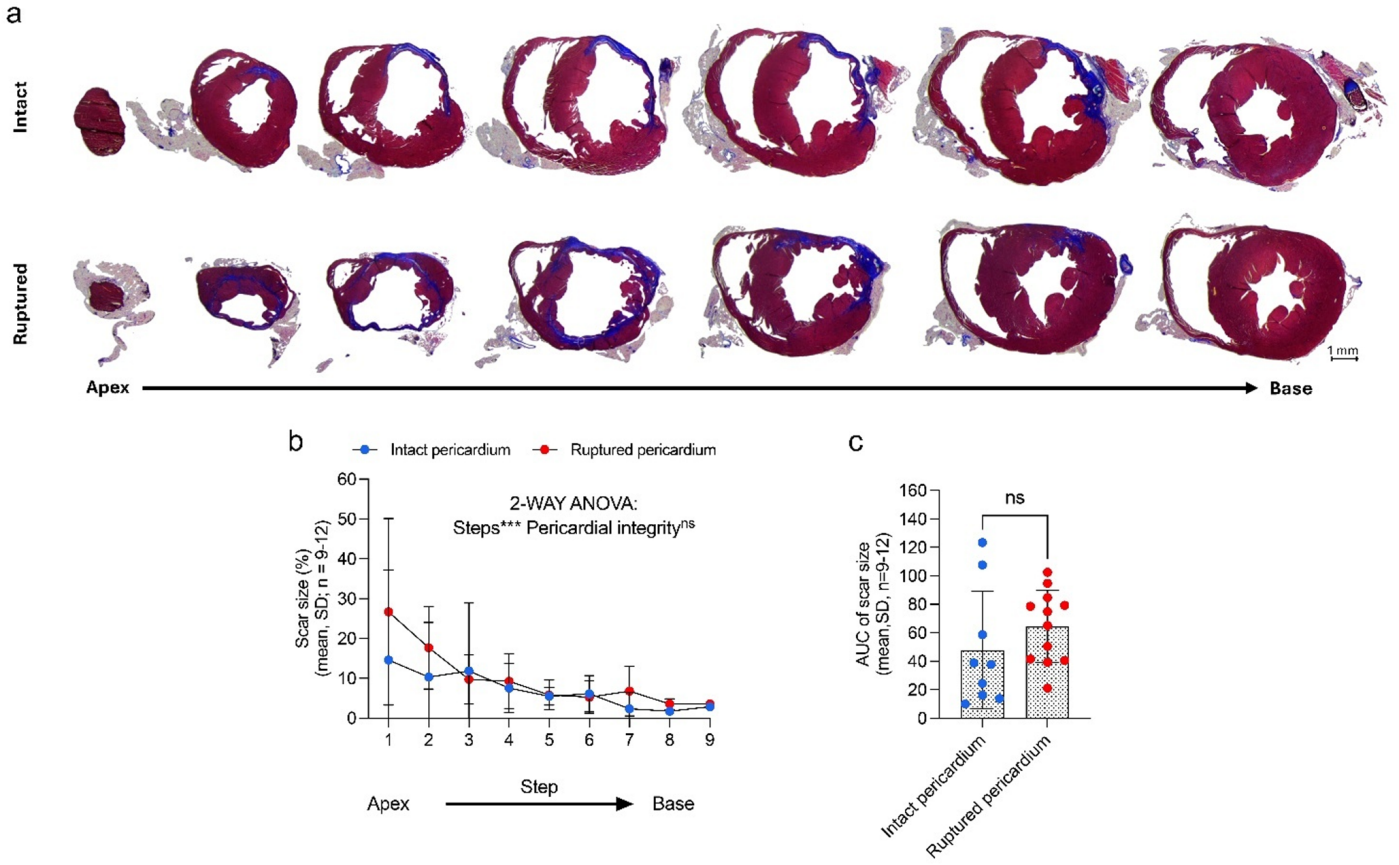
Assessment of myocardial scarring 10 weeks post-MI in mice with intact versus ruptured pericardium. (**a**) Representative examples of histological MT sections from both groups, from apex to base, (**b**) step‑wise distribution of scar size along the left ventricle, expressed as % collagen‑positive area of total myocardial area for each group, and (**c**) group-level total scar size, calculated for each heart as the area under the curve (AUC) of the section-wise values and shown as mean ± SD for each group. Statistics include two-way ANOVA with post hoc testing, ns: non-significant, ***: *p* ≤ 0.001

Finally, we assessed the impact of pericardial integrity on cardiac tissue remodeling as reflected by collagen accumulation and calculated as percentage scar size at 10 weeks post-MI (Fig. 5a + b). We observed no noticeable differences in the overall appearance of the hearts after fixation between the groups. Overall, histology demonstrated consistent scar morphology between MI hearts with intact- and ruptured pericardium, where extensive, frequently transmural fibrosis were observed, with variable LV wall thinning and chamber dilation throughout the hearts (Fig. 5a). Scar size was assessed across equally spaced steps from apex to base, and localization and the extent of scarring at each level was similar between hearts with intact- and ruptured pericardium (*p* < 0.05, Fig. 5b). As calculated from area under the curve (AUC) for each individual mice, the mean scar size was 47.9 ± 41.4% and 64.4 ± 25.4% (mean, SD, *n* = 9–12) for hearts with intact- and ruptured pericardium, respectively, with no difference between groups (*p* = 0.2716, Fig. 5c). These data suggest that infarct size is similar between groups of adult female mice and independent of pericardial integrity 10 weeks post-MI.

## Discussion

In this study, we used ultra-high-frequency in the real-time ultrasound system Vevo F2 to investigate the potential impact of pericardial disruption in the mouse LAD MI model as a surgical variable not present upon MI in humans. Overall, longitudinal changes in LV heart function, structural remodeling, and scaring after MI were comparable between mice with intact- and ruptured pericardium after MI, but MI hearts with a ruptured pericardium alone seem to exhibit a slightly difference in remodeling, which may indicate possible compensatory mechanism with posterior wall thickening and slightly increased EF and SV. This may have implications for using mice with LAD as a model for MI in humans, where the pericardium remains intact. Historically, the pericardium has received little attention, and resection of the pericardium in humans with constrictive pericarditis remains a suitable and safe treatment option [54] suggesting that the pericardium is non-critical to overall heart function under pathological conditions [54]. However, recent insight suggests that factors and cells in the pericardium indeed may contribute to remodeling after MI [6, 11, 12, 26], and pericardial rupture in the mouse MI LAD model may thus affect these processes and the results. Although the concept of pericardial restraint is well established in humans [55], the murine pericardium is far less restrictive, as it is thin and highly compliant in structure [56] as supposed to human pericardium. Therefore, any mechanical limitation of diastolic expansion must be modest in mice as compared to humans. It is evident that rupture of the pericardium provides better access and visibility to the LAD procedure, but at the same time it disrupts the functional role of the pericardium [57], its immune environment [25], and it may increase the likelihood of pericardial adhesions and inflammatory response [26]. Due to our method of preserving the pericardium, we cannot exclude the formation of pericardial adhesions during post-MI healing. However, during heart extraction post-fixation, we did not observe gross differences in heart appearance that would suggest overt mechanical restriction or dilation. Here, we extend the knowledge of pericardial integrity to LV functionality and -structure as well as scar size after MI in the adult mouse. The use herein of Vevo F2’s improved special- and temporal resolution increases confidence in our results showing no major impact on global systolic function upon pericardial rupture. Specifically, we chose the Modified biplane Simpson’s method in B-mode for measurements and calculations [33], which assumes geometric symmetry, but confers more accuracy than previous M-mode calculations [33]. However, the symmetry may be altered to some degree in MI hearts [46], depending on the location and extent of the scaring, and morphological changes of the heart wall following MI. In our assessments, the LV contours remained sufficiently traceable for reproducible quantification as suggested by others [40], and we thus did not apply the short-axis Simpson’s method using sequential parasternal short-axis images [58], which has been proposed as a flexible and reliable option for more severe ventricular remodeling [40]. Even so, our baseline data were consistent with reported normal values for FS [33, 49] and EF [50, 51] in adult mice under the same anesthesia, which supports both the accuracy of our acquisition and the reliability of the Modified biplane Simpson’s method in detecting post-MI changes [40] in our MI model. Our data showed that all mice, regardless of pericardial disruption, had impaired heart function. The ESV increase already observed 1 week after MI is of particular interest as it indicates that the damaged heart muscle is not able to contract as effectively, leading to larger amounts of blood remaining in the ventricle at the end of contraction [59]. This closely mirrors clinical observations in humans, where elevated ESV after exercise independently predicts mortality in patients with coronary heart disease [60]. Furthermore, increased ESV after MI is a hallmark of adverse ventricular remodeling and has been reported as a stronger predictor of future cardiovascular events than EF or EDV alone [59]. ESV is a load-independent marker of contractility and remodeling, underscoring that even modest changes can carry prognostic weight across species. In our model, the early elevated ESV likely reflects an early trajectory toward heart failure [60], thereby highlighting the model fidelity in reproducing clinically relevant injury. Moreover, we did observe an interesting difference in LVPW thickness between MI hearts with intact- and ruptured pericardium, whereas the LVAW remained similar. Increases in LVPW often reflect a compensatory LV hypertrophy introduced by sustained heart stress such as MI [61]. The LVAW or -PW thicknesses are measured at the mid-papillary muscle level, to assess structural changes post-MI [35]. Taking into consideration that the observed MI´s were predominantly located towards the apex, and not as widespread towards the base of the ventricle, thinning of the LVAW may have been neglected due to the location of the measurements [9]. Nevertheless, the mildly increased systolic LVPW for MI hearts with ruptured pericardium observed both at week 1 and 10 may explain the observed increase in EF and SV at 10 weeks post-MI. This increase in EF should not be interpreted as improved systolic performance, as EF is load-dependent. Indeed, the parallel rise in ESV and SV indicates altered loading and remodeling rather than enhanced contractility [62, 63]. Thus, our data most likely suggest a decline in cardiac function. However, whether this pattern represents a transient adaptation or the beginning of a maladaptive remodeling remains unknown and would require longer-term mechanistic studies in the future. In humans, the fibrous pericardium provides meaningful mechanical restraint, and its removal can increase diastolic filling [55]. While pericardial rupture in our model may alter the local mechanical or inflammatory environment and could influence remodeling, substantial restriction of diastolic filling by an intact murine pericardium seems less likely. Differences between groups are therefore more plausibly related to remodeling dynamics and subtle environmental changes rather than a pronounced pericardial constraint, which distinguishes the mouse model from the human clinical situation. Importantly, an increased EF is not inherently detrimental, but rather, the altered pericardial environment following rupture may impose additional mechanical or inflammatory stress that could influence the long-term remodeling outcomes [64, 65]. Yet, our data may suggest that rupturing the pericardium provides an additional variable of stress to the heart where long-term prognosis may be worse as compared to the scenario of intact pericardium upon MI. In cardiovascular research exploiting the mouse LAD-ligation MI model, one should thus consider that rupturing the pericardium may alter the pattern of remodeling and compensation, potentially enforcing some artefactual concerns not applicable to the human scenario of MI. Notably, we used female mice for simplicity, to assess the effects of pericardial rupture on post‑MI remodeling. Yet, as male mice typically exhibit more pronounced adverse remodeling after MI [66], the magnitude of some of the observed effects may differ in males. Thus, future studies including both sexes will be essential to determine whether the remodeling patterns associated with pericardial rupture are shared or diverge between males and females, to better define their translational relevance.

### Limitations

Besides limitations already mentioned above, it is important to consider the difficulty of the LAD technique. Without opening the pericardium, the suture is placed without clear visualization of the LAD, leading to less control of the suture placement and the infarct location and size. Although we did not observe a difference in the success of MI introduction or animal survival, the technical complexity of LAD is a clear limitation and known factor for variability of scar size etc [17]. Moreover, despite the advanced Vevo F2 system reduces the probability of measuring false negatives parameters reflecting cardiac dysfunction, it does not fully exclude subtle regional wall-motion changes not captured by EF [67]. Finally, it is important to note that the anesthesia used in this study (ketamine-xylazine) is less commonly used for echocardiography in mice [68], and it has been shown to have depressant effects on heart rate in mice [69]. This may limit the comparability with studies using inhalational anesthesia (e.g. isoflurane) [70] and should be considered when evaluating the functional outcomes herein with other studies. Finally, as discussed above, our observations apply to female mice as used herein, and we can only speculate on the response in male mice.

## Conclusion

In conclusion, our data indicate that pericardial integrity does not universally alter heart function and scar size following LAD ligation in adult female mice, but may have some previously unnoticed compensatory effects not present after MI in man and that should be considered when modeling MI in the mouse.

## Supplementary Information

Below is the link to the electronic supplementary material.

Supplementary Material 1

## Data Availability

All data supporting the findings of this study are available within the paper and its Supplementary Information.

## References

[1] Roth GA, Mensah GA, Johnson CO et al (2020) Global Burden of Cardiovascular Diseases and Risk Factors, 1990–2019: Update From the GBD 2019 Study. J Am Coll Cardiol 76:2982–3021. 10.1016/j.jacc.2020.11.010 33309175 10.1016/j.jacc.2020.11.010 PMC7755038

[2] Naghavi M, Ong KL, Aali A et al (2024) Global burden of 288 causes of death and life expectancy decomposition in 204 countries and territories and 811 subnational locations, 1990–2021: a systematic analysis for the Global Burden of Disease Study 2021. Lancet, 403(10440):2100–2132. 10.1016/s0140-6736(24)00367-2 10.1016/S0140-6736(24)00367-2 PMC11126520 38582094

[3] Liu Y, Li L, Wang Z, Zhang J, Zhou Z (2023) Myocardial ischemia-reperfusion injury; Molecular mechanisms and prevention. Microvasc Res 149:104565. 10.1016/j.mvr.2023.104565 37307911 10.1016/j.mvr.2023.104565

[4] Sagris M, Antonopoulos AS, Theofilis P et al (2022) Risk factors profile of young and older patients with myocardial infarction. Cardiovasc Res 118(10):2281–2292. 10.1093/cvr/cvab264 34358302 10.1093/cvr/cvab264

[5] Salari N, Morddarvanjoghi F, Abdolmaleki A et al (2023) The global prevalence of myocardial infarction: a systematic review and meta-analysis. BMC Cardiovasc Disord 23(1):206. 10.1186/s12872-023-03231-w 37087452 10.1186/s12872-023-03231-w PMC10122825

[6] Fang M, Xiang FL, Braitsch CM, Yutzey KE (2016) Epicardium-derived fibroblasts in heart development and disease. J Mol Cell Cardiol 91:23–27. 10.1016/j.yjmcc.2015.12.019 26718723 10.1016/j.yjmcc.2015.12.019 PMC4764446

[7] Humeres C, Frangogiannis NG (2019) Fibroblasts in the infarcted, remodeling, and, failing heart. JACC Basic Transl Sci 4(3):449–467. 10.1016/j.jacbts.2019.02.006 31312768 10.1016/j.jacbts.2019.02.006 PMC6610002

[8] Richardson WJ, Holmes JW (2015) Why is infarct expansion such an elusive therapeutic target? J Cardiovasc Transl Res 8(7):421–430. 10.1007/s12265-015-9652-2 26390882 10.1007/s12265-015-9652-2 PMC4846979

[9] Frantz S, Hundertmark MJ, Schulz-Menger J, Bengel FM, Bauersachs J (2022) Left ventricular remodelling post-myocardial infarction: pathophysiology, imaging, and novel therapies. Eur Heart J 43(27):2549–2561. 10.1093/eurheartj/ehac223 35511857 10.1093/eurheartj/ehac223 PMC9336586

[10] Jaworska-Wilczynska M, Trzaskoma P, Szczepankiewicz AA, Hryniewiecki T (2016) Pericardium: structure and function in health and disease. Folia Histochem Cytobiol 54(3):121–125. 10.5603/FHC.a2016.0014 27654013 10.5603/FHC.a2016.0014

[11] Ruiz-Villalba A, Simon AM, Pogontke C et al (2015) Interacting resident epicardium-derived fibroblasts and recruited bone marrow cells form myocardial infarction scar. J Am Coll Cardiol 65:2057–2066. 10.1016/j.jacc.2015.03.520 25975467 10.1016/j.jacc.2015.03.520

[12] Jensen CH, Johnsen RH, Eskildsen T et al (2024) Pericardial delta like non-canonical NOTCH ligand 1 (Dlk1) augments fibrosis in the heart through epithelial to mesenchymal transition. Clin Transl Med 14(2):e1565. 10.1002/ctm2.1565 38328889 10.1002/ctm2.1565 PMC10851088

[13] LindseyML, Brunt KR, Kirk JA et al (2021) Guidelines for in vivo mouse models of myocardial infarction. Am J Physiol Heart Circ Physiol 321(6): pp. H1056-h1073 10.1152/ajpheart.00459.2021 10.1152/ajpheart.00459.2021 PMC8834230 34623181

[14] De Villiers C, Riley PR (2020) Mouse models of myocardial infarction: comparing permanent ligation and ischaemia-reperfusion. Dis Model Mech 13:11. 10.1242/dmm.046565 10.1242/dmm.046565 PMC7687859 33361140

[15] von Scheidt M, Zhao Y, Kurt Z et al (2017) Applications and limitations of mouse models for understanding human atherosclerosis. Cell Metab 25(2):248–261. 10.1016/j.cmet.2016.11.001 27916529 10.1016/j.cmet.2016.11.001 PMC5484632

[16] Martin TP, MacDonald EA, Elbassioni AAM et al (2022) Preclinical models of myocardial infarction: from mechanism to translation. Br J Pharmacol 179(5):770–791. 10.1111/bph.15595 34131903 10.1111/bph.15595

[17] Bassat E, Perez DE, Tzahor E (2021) Myocardial infarction techniques in adult mice. Methods Mol Biol 2158:3–21. 10.1007/978-1-0716-0668-1_1 32857361 10.1007/978-1-0716-0668-1_1

[18] He Y, Pan X, Liu Z et al (2025) METTL3 silencing suppresses cardiac fibrosis post myocardial infarction via m6A modification of SMOC2. J Cell Mol Med 29(17):e70829. 10.1111/jcmm.70829 40913254 10.1111/jcmm.70829 PMC12413310

[19] Guo CH, Wang QQ, Li JQ et al (2025) BMP1 inhibitor UK383367 improves MI-induced cardiac remodeling and fibrosis in mice via ameliorating macrophage polarization and mitochondrial dysfunction. Acta Pharmacol Sin. 10.1038/s41401-025-01655-y 40897852 10.1038/s41401-025-01655-y PMC12811289

[20] Chang T, Jin Y, Fan C et al (2025) N-acetylglucosaminyltransferase V attenuates myocardial infarction by mediating the insulin-like growth factor 1 receptor signaling pathway. J Transl Int Med 13(3):281–294. 10.1515/jtim-2025-0021 40896287 10.1515/jtim-2025-0021 PMC12392078

[21] Wang M, Zhao C, Li T et al (2025) Hypoxia-conditioned cardiomyocyte-derived exosomes attenuate myocardial injury via ANP-mediated M2 macrophage polarization. Gen Physiol Biophys 44(5):377–389. 10.4149/gpb_2025022 40923657 10.4149/gpb_2025022

[22] Feng J, Li Y, Li Y et al (2024) Versican promotes cardiomyocyte proliferation and cardiac repair. Circulation 149(13):1004–1015. 10.1161/circulationaha.123.066298 37886839 10.1161/CIRCULATIONAHA.123.066298

[23] Li J, Sun S, Zhu D et al (2024) Inhalable stem cell exosomes promote heart repair after myocardial infarction. Circulation 150(9):710–723. 10.1161/circulationaha.123.065005 39186525 10.1161/CIRCULATIONAHA.123.065005 PMC11349039

[24] Ahn D, Cheng L, Moon C, Spurgeon H, Lakatta EG, Talan MI (2004) Induction of myocardial infarcts of a predictable size and location by branch pattern probability-assisted coronary ligation in C57BL/6 mice. Am J Physiol Heart Circ Physiol 286(3):H1201–H1207. 10.1152/ajpheart.00862.2003 14766681 10.1152/ajpheart.00862.2003

[25] Isidoro CA, Deniset JF (2023) Pericardial immune cells and their evolving role in cardiovascular pathophysiology. Can J Cardiol 39(8):1078–1089. 10.1016/j.cjca.2023.05.017 37270165 10.1016/j.cjca.2023.05.017

[26] Deniset JF, Belke D, Lee WY et al (2019) Gata6(+) pericardial cavity macrophages relocate to the injured heart and prevent cardiac fibrosis. Immunity 51(1):131–140e5. 10.1016/j.immuni.2019.06.010 31315031 10.1016/j.immuni.2019.06.010 PMC7574643

[27] Yu X, Newland SA, Zhao TX et al (2021) Innate Lymphoid Cells Promote Recovery of Ventricular Function After Myocardial Infarction. J Am Coll Cardiol 78(11):1127–1142. 10.1016/j.jacc.2021.07.018 34503682 10.1016/j.jacc.2021.07.018 PMC8434674

[28] Fatehi Hassanabad A, Belke DD, Turnbull J et al (2021) An intact pericardium ischemic rodent model. J Vis Exp. 10.3791/62720 34542537 10.3791/62720

[29] Horckmans M, Bianchini M, Santovito D et al (2018) Pericardial adipose tissue regulates granulopoiesis, fibrosis, and cardiac function after myocardial infarction. Circulation 137(9):948–960. 10.1161/circulationaha.117.028833 29167227 10.1161/CIRCULATIONAHA.117.028833

[30] Mylonas K, Jackson-Jones L, Andrews J et al (2019) The pericardium promotes cardiac repair and remodelling post-myocardial infarction

[31] Jin H, Liu K, Huang X et al (2022) Genetic Lineage Tracing of Pericardial Cavity Macrophages in the Injured Heart. Circ Res 130(11):1682–1697. 10.1161/circresaha.122.320567 35440174 10.1161/CIRCRESAHA.122.320567

[32] Salerno N, Di Costanzo A, Marino F et al (2025) Echocardiographic Assessment of Cardiac Function in Mouse Models of Heart Disease. Int J Mol Sci 26:13. 10.3390/ijms26135995 10.3390/ijms26135995 PMC12249879 40649774

[33] Gao S, Ho D, Vatner DE, Vatner SF (2011) Echocardiography in Mice. Curr Protoc Mouse Biol 1:71–83. 10.1002/9780470942390.mo100130 21743841 10.1002/9780470942390.mo100130 PMC3130310

[34] Gao XM, Dart AM, Dewar E, Jennings G, Du XJ (2000) Serial echocardiographic assessment of left ventricular dimensions and function after myocardial infarction in mice. Cardiovasc Res 45(2):330–338. 10.1016/s0008-6363(99)00274-6 10728353 10.1016/s0008-6363(99)00274-6

[35] Guide to Small Animal Basic Echocardiography using the Vevo Ultrasound Systems. (2023) visualsonics.com: FUJIFILM VisualSonics, Inc. https://www.visualsonics.com/sites/default/files/MKT03791%20Guide%20to%20Basic%20Echocardiography%20Rev%201.0.pdf

[36] Salvas JP, Moore-Morris T, Goergen CJ, Sicard P (2025) Left atrial reservoir strain as a predictor of cardiac dysfunction in a murine model of pressure overload. Acta Physiol (Oxf) 241(2):e14277. 10.1111/apha.14277 39822162 10.1111/apha.14277 PMC11737473

[37] Prendiville TW, Ma Q, Lin Z, Zhou P, He A, Pu WT (2014) Ultrasound-guided transthoracic intramyocardial injection in mice. J Vis Exp no. 90:e51566. 10.3791/51566 10.3791/51566 PMC4267063 25146757

[38] Kustiati U, Widayati WT, Kusindarta DL et al (2025) A lung cancer mouse model system based on an inbred C3H strain: Ultrasound imaging, pathological analysis, and proteomic biomarker identification. Vet World 18(5):1101–1108. 10.14202/vetworld.2025.1101-1108 40584122 10.14202/vetworld.2025.1101-1108 PMC12205233

[39] Todd EA, Williams M, Kamiar A, Rasmussen MA, Shehadeh LA (2022) Echocardiography protocol: A tool for infrequently used parameters in mice. Front Cardiovasc Med 9:1038385. 10.3389/fcvm.2022.1038385 36620641 10.3389/fcvm.2022.1038385 PMC9810757

[40] Lindsey ML, Kassiri Z, Virag JAI, de Castro Brás LE, Scherrer-Crosbie M (2018) Guidelines for measuring cardiac physiology in mice. Am J Physiol Heart Circ Physiol 314(4):H733–h752. 10.1152/ajpheart.00339.2017 29351456 10.1152/ajpheart.00339.2017 PMC5966769

[41] Zacchigna S, Paldino A, Falcão-Pires I et al (2021) Towards standardization of echocardiography for the evaluation of left ventricular function in adult rodents: a position paper of the ESC Working Group on Myocardial Function. Cardiovasc Res 117(1):43–59. 10.1093/cvr/cvaa110 32365197 10.1093/cvr/cvaa110

[42] Schiller NB, Acquatella H, Ports TA et al (1979) Left ventricular volume from paired biplane two-dimensional echocardiography. Circulation 60(3):547–555. 10.1161/01.cir.60.3.547 455617 10.1161/01.cir.60.3.547

[43] Scherrer-Crosbie M, Thibault HB (2008) Echocardiography in translational research: of mice and men. J Am Soc Echocardiogr 21(10):1083–1092. 10.1016/j.echo.2008.07.001 18723318 10.1016/j.echo.2008.07.001 PMC2648388

[44] Gissler MC, Antiochos P, Ge Y, Heydari B, Gräni C, Kwong RY (2024) Cardiac magnetic resonance evaluation of LV remodeling post-myocardial infarction: prognosis, monitoring and trial endpoints. JACC Cardiovasc Imaging 17(11):1366–1380. 10.1016/j.jcmg.2024.03.012 38819335 10.1016/j.jcmg.2024.03.012

[45] Zacchigna S, Paldino A, Falcão-Pires I et al (2020) Towards standardization of echocardiography for the evaluation of left ventricular function in adult rodents: a position paper of the ESC Working Group on Myocardial Function. Cardiovascular Res 117(1):43–59. 10.1093/cvr/cvaa110 10.1093/cvr/cvaa110 32365197

[46] Salerno N, Di Costanzo A, Marino F et al (2025) Echocardiographic assessment of cardiac function in mouse models of heart disease. Int J Mol Sci 26(13):5995. https://www.mdpi.com/1422-0067/26/13/5995 40649774 10.3390/ijms26135995 PMC12249879

[47] Takagawa J, Zhang Y, Wong ML et al (2007) Myocardial infarct size measurement in the mouse chronic infarction model: comparison of area- and length-based approaches. J Appl Physiol 102(6):2104–2111. 10.1152/japplphysiol.00033.2007 17347379 10.1152/japplphysiol.00033.2007 PMC2675697

[48] (2003) Malocclusion in the laboratory mouse, JAX^®^ NOTES, no. 489. https://research.uky.edu/division-laboratory-animal-resources/malocclusion-laboratory-mouse

[49] Yang XP, Liu YH, Rhaleb NE, Kurihara N, Kim HE, Carretero OA (1999) Echocardiographic assessment of cardiac function in conscious and anesthetized mice. Am J Physiol 277(5). H1967-74 10.1152/ajpheart.1999.277.5.H1967 10.1152/ajpheart.1999.277.5.H1967 10564153

[50] Vinhas M, Araújo AC, Ribeiro S, Rosário LB, Belo JA (2013) Transthoracic echocardiography reference values in juvenile and adult 129/Sv mice. Cardiovasc Ultrasound 11:12. 10.1186/1476-7120-11-12 23634975 10.1186/1476-7120-11-12 PMC3651272

[51] Stypmann J, Engelen MA, Troatz C, Rothenburger M, Eckardt L, Tiemann K (2009) Echocardiographic assessment of global left ventricular function in mice. Lab Anim 43(2):127–137. 10.1258/la.2007.06001e 19237453 10.1258/la.2007.06001e

[52] Leancă SA, Crișu D, Petriș AO et al (2022) Left ventricular remodeling after myocardial infarction: from physiopathology to treatment. Life 12(8):1111. https://www.mdpi.com/2075-1729/12/8/1111 35892913 10.3390/life12081111 PMC9332014

[53] Kanno S, Lerner DL, Schuessler RB et al (2002) Echocardiographic evaluation of ventricular remodeling in a mouse model of myocardial infarction. J Am Soc Echocardiogr 15(6):601–609. 10.1067/mje.2002.117560 12050601 10.1067/mje.2002.117560

[54] Fardman A, Charron P, Imazio M, Adler Y (2016) European guidelines on pericardial diseases: a focused review of novel aspects. Curr Cardiol Rep 18(5):46. 10.1007/s11886-016-0721-1 27007597 10.1007/s11886-016-0721-1

[55] Borlaug BA, Reddy YNV (2019) The role of the pericardium in heart failure: implications for pathophysiology and treatment. JACC Heart Fail 7(7):574–585. 10.1016/j.jchf.2019.03.021 31248569 10.1016/j.jchf.2019.03.021 PMC6601642

[56] Kojima A, Sakaue T, Okazaki M et al (2019) A simple mouse model of pericardial adhesions. J Cardiothorac Surg 14(1):124. 10.1186/s13019-019-0940-9 31253183 10.1186/s13019-019-0940-9 PMC6599257

[57] Janicki JS, Weber KT (1980) The pericardium and ventricular interaction, distensibility, and function. Am J Physiol 238(4):H494–503. 10.1152/ajpheart.1980.238.4.H494 7377320 10.1152/ajpheart.1980.238.4.H494

[58] Russo I, Micotti E, Fumagalli F et al (2019) A novel echocardiographic method closely agrees with cardiac magnetic resonance in the assessment of left ventricular function in infarcted mice. Sci Rep 9(1):3580. 10.1038/s41598-019-40393-0 30837662 10.1038/s41598-019-40393-0 PMC6400943

[59] Cohn JN, Ferrari R, Sharpe N (2000) Cardiac remodeling–concepts and clinical implications: a consensus paper from an international forum on cardiac remodeling. Behalf of an International Forum on Cardiac Remodeling. J Am Coll Cardiol 35(3):569–582. 10.1016/s0735-1097(99)00630-0 10716457 10.1016/s0735-1097(99)00630-0

[60] Turakhia MP, McManus DD, Whooley MA, Schiller NB (2009) Increase in end-systolic volume after exercise independently predicts mortality in patients with coronary heart disease: data from the Heart and Soul Study. Eur Heart J 30(20):2478–2484. 10.1093/eurheartj/ehp270 19578167 10.1093/eurheartj/ehp270 PMC2761597

[61] Bhumitrakul JB, Osotthanakorn TO, Chantranuwat PC, Wicheantawatchai AW, Boonyaratavej SB, Puwanant SP (2024) Left ventricular hypertrophy, wall thickness, and histological myocyte alteration in end-stage heart failure: a call for a standard definition. Eur Heart J 45. 10.1093/eurheartj/ehae666.096.no.Supplement_1

[62] Protti I, van den Enden A, Van Mieghem NM, Meuwese CL, Meani P (2024) Looking back, going forward: understanding cardiac pathophysiology from pressure-volume loops. Biology (Basel) 13(1). 10.3390/biology13010055 10.3390/biology13010055 PMC10813445 38275731

[63] Katz AM, Rolett EL (2016) Heart failure: when form fails to follow function. Eur Heart J 37(5):449–454. 10.1093/eurheartj/ehv548 26497163 10.1093/eurheartj/ehv548

[64] Sutton MG, Sharpe N (2000) Left ventricular remodeling after myocardial infarction: pathophysiology and therapy. Circulation 101(25):2981–2988. 10.1161/01.cir.101.25.2981 10869273 10.1161/01.cir.101.25.2981

[65] Leancă SA, Crișu D, Petriș AO et al (2022) Left ventricular remodeling after myocardial infarction: from physiopathology to treatment. Life (Basel) 12(8). 10.3390/life12081111 10.3390/life12081111 PMC9332014 35892913

[66] Wu JC, Nasseri BA, Bloch KD, Picard MH, Scherrer-Crosbie M (2003) Influence of sex on ventricular remodeling after myocardial infarction in mice. J Am Soc Echocardiogr 16(11):1158–1162. 10.1067/s0894-7317(03)00648-5 14608287 10.1067/S0894-7317(03)00648-5

[67] Zhang TY, Zhao BJ, Wang T, Wang J (2021) Effect of aging and sex on cardiovascular structure and function in wildtype mice assessed with echocardiography. Sci Rep 11(1):22800. 10.1038/s41598-021-02196-0 34815485 10.1038/s41598-021-02196-0 PMC8611093

[68] Pachon RE, Scharf BA, Vatner DE, Vatner SF (2015) Best anesthetics for assessing left ventricular systolic function by echocardiography in mice. Am J Physiol Heart Circ Physiol 308(12):H1525–H1529. 10.1152/ajpheart.00890.2014 25862835 10.1152/ajpheart.00890.2014 PMC4469873

[69] Virgilio T, Latino I, Pizzichetti C et al (2025) Impact of prolonged isoflurane or ketamine–xylazine anesthesia with or without buprenorphine and oxygen on mouse vitals and immune responses. Lab Anim 54(10):270–277. 10.1038/s41684-025-01614-4 10.1038/s41684-025-01614-4 PMC12484078 40968194

[70] Kawahara Y, Tanonaka K, Daicho T et al (2005) Preferable anesthetic conditions for echocardiographic determination of murine cardiac function. J Pharmacol Sci 99(1):95–104. 10.1254/jphs.FP0050343 16177543 10.1254/jphs.fp0050343

