# Supplementary material for: The impact of pericardial disruption on heart function and remodeling after myocardial infarction in mice
Source: Int J Cardiovasc Imaging. 2026 Mar 21;42(7):1381–94. doi: 10.1007/s10554-026-03688-8 (PMC13375912; doi:10.1007/s10554-026-03688-8)
Supplement: Supplementary file 1 — Supplementary Material 1 [file 10554_2026_3688_MOESM1_ESM.pdf]

Title:

The impact of pericardial disruption on heart function and remodeling after myocardial infarction in mice.

Journal: The International Journal of Cardiovascular Imaging

Authors:

Sara Munk Laursen[1,2#], Ditte Gry Ellman[1,2#], Charlotte Harken Jensen[1,2], Ditte Caroline Andersen[1,2*]

[1]Andersen Group, Dep. of Clinical Biochemistry, Odense University Hospital, Denmark.

[2]Clinical Institute, University of Southern Denmark, Odense, Denmark.

[*]Corresponding author: Ditte Caroline Andersen, E-mail: dandersen@health.sdu.dk

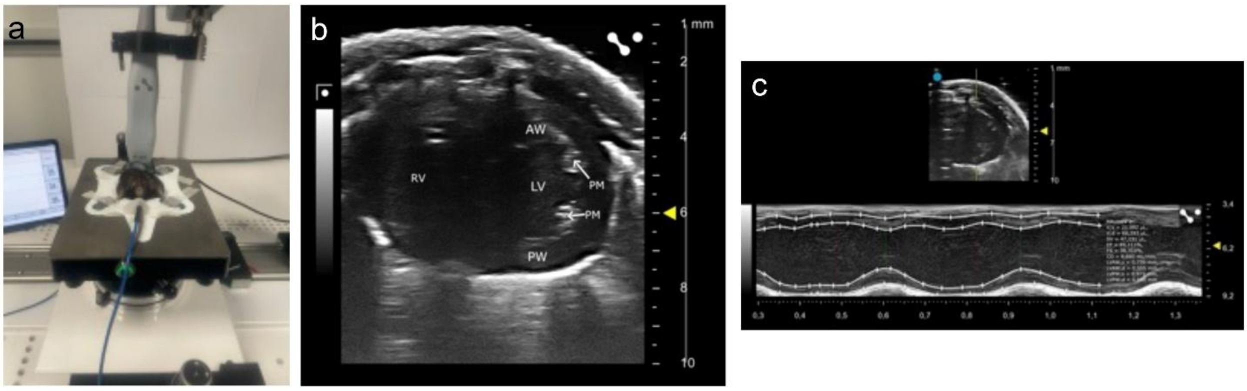

Online Resource 1 Experimental setup for murine echocardiography in PSSAX view, B- and M-mode, with representative images and measurements. (**a**) Mouse in supine position on a pre-warmed platform with thorax shaved for scanning and paws connected to monitoring electrodes and transducer positioning for PSSAX view. (**b**) Representative images of B-mode image of the left ventricle in PSSAX view, and (**c**) M-mode image with left ventricular measurements in PSSAX view at mid-papillary level. LV: left ventricle, RV: right ventricle, AW: anterior wall, PW: posterior wall, PM: papillary muscles, PSSAX: parasternal short axis

Online Resource 2 Collected data of measurements (mean, SD) and sample sizes (n) obtained from each group of EF, FS, and LV Volume values (in end-systole and end-diastole) in all views and modes (PSLAX and PSSAX view, B- and M-mode imaging). LV: left ventricle, EF: ejection fraction, FS: fractional shortening, PSLAX: parasternal long axis, PSSAX: parasternal short axis

	Intact pericardium			Open pericardium		
	Mean	SD	n	Mean	SD	n
**EF – Long axis – M-mode**						
Baseline	62.231	4.474	9	62.820	4.646	12
1 week	49.932	6.350	9	49.720	8.177	12
10 weeks	48.500	10.113	9	48.792	9.233	12
**EF – Long axis – B-mode**						
Baseline	55.889	5.004	9	55.966	4.301	12
1 week	43.588	5.854	9	38.756	8.561	12
10 weeks	46.831	7.358	9	49.321	9.965	12
**EF – Short-axis – M-mode**						
Baseline	61.429	3.482	9	62.047	4.054	12
1 week	52.409	8.847	9	54.323	5.685	12
10 weeks	46.285	9.576	9	43.871	8.048	12
**FS – Long axis – M-mode**						
Baseline	33.076	3.265	9	33.499	3.321	12
1 week	23.985	3.149	7	24.955	4.615	11
10 weeks	24.667	6.251	9	24.716	5.638	12
**FS – Short-axis – M-mode**						
Baseline	32.460	2.432	9	32.878	2.930	12
1 week	25.398	4.815	7	28.912	3.794	11
10 weeks	23.128	5.766	9	21.700	4.595	12
**EDV – Long-axis – M-mode**						
Baseline	62.433	8.878	9	60.927	7.046	12
1 week	77.752	9.913	8	75.711	17.045	12
10 weeks	94.990	34.867	9	92.392	22.156	12
**EDV – Long-axis – B-mode**						
Baseline	51.842	6.116	9	52.961	4.352	12
1 week	60.455	16.258	9	62.289	10.283	12
10 weeks	65.559	17.160	9	70.668	18.925	12
**EDV – Short-axis – M-mode**						
Baseline	63.446	5.565	9	60.423	5.815	12
1 week	80.764	13.983	9	77.196	22.166	12
10 weeks	86.624	36.795	9	86.617	15.963	12
**ESV – Long-axis – M-mode**						
Baseline	23.861	5.346	9	22.770	4.667	12
1 week	39.562	7.730	8	38.344	11.908	12
10 weeks	51.293	27.887	9	48.952	19.529	12
**ESV – Long-axis – B-mode**						
Baseline	23.048	4.995	9	23.440	3.821	12
1 week	34.687	12.649	9	38.362	9.064	12
10 weeks	35.711	12.831	9	37.035	14.917	12
**ESV – Short-axis – M-mode**						
Baseline	23.861	5.346	9	22.770	4.667	12
1 week	39.562	7.730	8	38.344	11.908	12
10 weeks	51.293	27.887	9	48.952	19.529	12
